# Global Proteomic Profiling of *Piscirickettsia* *salmonis* and Salmon Macrophage-Like Cells during Intracellular Infection

**DOI:** 10.3390/microorganisms8121845

**Published:** 2020-11-24

**Authors:** Javiera Ortiz-Severín, Dante Travisany, Alejandro Maass, Verónica Cambiazo, Francisco P. Chávez

**Affiliations:** 1 Laboratorio de Microbiología de Sistemas, Departamento de Biología, Facultad de Ciencias, Universidad de Chile, Santiago 7800003, Chile; javiera.o.s@gmail.com; 2 Laboratorio de Bioinformática y Expresión Génica, Instituto de Nutrición y Tecnología de los Alimentos, Universidad de Chile, Santiago 7830490, Chile; vcambiaz@inta.uchile.cl; 3 Fondap Center for Genome Regulation (Fondap 15090007), Universidad de Chile, Santiago 8370415, Chile; dtravisany@gmail.com (D.T.); amaass@dim.uchile.cl (A.M.); 4 Centro de Modelamiento Matemático (AFB170001) and Departamento de Ingeniería Matemática, Facultad de Ciencias Físicas y Matemáticas, Universidad de Chile and UMI-CNRS 2807, Santiago 8370415, Chile

**Keywords:** fish pathogen, host–pathogen interaction, proteomics, infection assays, virulence factors

## Abstract

*Piscirickettsiasalmonis* is an intracellular bacterial fish pathogen that causes piscirickettsiosis, a disease with numerous negative impacts in the Chilean salmon farming industry. Although transcriptomic studies of *P. salmonis* and its host have been performed, dual host–pathogen proteomic approaches during infection are still missing. Considering that gene expression does not always correspond with observed phenotype, and bacteriological culture studies inadequately reflect infection conditions, to improve the existing knowledge for the pathogenicity of *P. salmonis*, we present here a global proteomic profiling of *Salmon salar* macrophage-like cell cultures infected with *P. salmonis* LF-89. The proteomic analyses identified several *P. salmonis* proteins from two temporally different stages of macrophages infection, some of them related to key functions for bacterial survival in other intracellular pathogens. Metabolic differences were observed in early-stage infection bacteria, compared to late-stage infections. Virulence factors related to membrane, lipopolysaccharide (LPS) and surface component modifications, cell motility, toxins, and secretion systems also varied between the infection stages. Pilus proteins, beta-hemolysin, and the type VI secretion system (T6SS) were characteristic of the early-infection stage, while fimbria, upregulation of 10 toxins or effector proteins, and the Dot/Icm type IV secretion system (T4SS) were representative of the late-infection stage bacteria. Previously described virulence-related genes in *P. salmonis* plasmids were identified by proteomic assays during infection in SHK-1 cells, accompanied by an increase of mobile-related elements. By comparing the infected and un-infected proteome of SHK-1 cells, we observed changes in cellular and redox homeostasis; innate immune response; microtubules and actin cytoskeleton organization and dynamics; alteration in phagosome components, iron transport, and metabolism; and amino acids, nucleoside, and nucleotide metabolism, together with an overall energy and ATP production alteration. Our global proteomic profiling and the current knowledge of the *P. salmonis* infection process allowed us to propose a model of the macrophage–*P. salmonis* interaction.

## 1. Introduction

Microbial infections are characterized by a constant interplay between pathogen and host, with pathogens exploiting various host functions during infection and hosts reacting with appropriate defense responses [1]. Therefore, understanding host–pathogen interaction is crucial for the development of effective vaccines and therapies [2].

Piscirickettsiosis or Salmonid Rickettsial Septicaemia (SRS) is one of the most threatening bacterial diseases in the Chilean salmon industry [3]. *Piscirickettsia salmonis*, the causative agent of SRS, has the ability to infect, survive, and replicate in salmonid monocyte/macrophage cell lines [4]. However, despite current advances in aquaculture infectious diseases, *P. salmonis* pathogenesis is still poorly understood, with many relevant aspects of its life cycle, virulence factors, and pathogenesis that remain to be elucidated.

A significant challenge for studying intracellular bacterial pathogens is understanding the molecular bases of disease development during host infection. Therefore, knowledge about bacterial virulence factors expressed during infection is crucial to gain a quantitative view of the pathogenic functions. Considering the scarce information existent to generate *P. salmonis* knockout mutants [5], and that molecular biology tools to manipulate *P. salmonis* genetic material are limited [6], omics approaches have emerged as effective tools in basic and applied research for the study of biological pathways involved in pathogen replication, host response, and disease progression.

Functional genomic studies of *P. salmonis* infection in salmon cells and tissues have been mainly focused on changes in gene expression following bacterial infection [7,8,9,10]. Particularly, transcriptomic analyses have provided evidence of host cellular processes and particular genes targeted during *P. salmonis* infection to promote intracellular survival and replication [7,11,12,13]. These analyses contributed to a better understanding of the in vivo infection process, since genes expressed in this condition can reveal the pathogen’s survival strategy in the intracellular environment. Recently, the simultaneous sequencing of both pathogen and host transcriptomes (dual transcriptomics) during the infection has uncover the complexity of the host–pathogen interactions. This global transcriptomic analysis was used to simultaneously analyze the transcriptome of *P. salmonis* during *Salmo salar* infection, revealing a bacterial dependency to host metabolism and nutrients accessibility [14].

The combination of proteomics with other omics approaches has expanded the repertoire of tools to study pathogen infections [15]. Identification of these host–pathogen protein interactions in the context of infection can be critical to understand the biology of infection and to discover novel targets for treatments against microbial pathogens. In *P. salmonis*, proteomics studies of cell-free growth conditions have been reported [16,17], including proteomic studies of *P. salmonis* extracellular vesicles during infection that contained proteins related to key functions for pathogen survival and plasmid-encoded toxins [18,19]. However, global proteomic profiling of host–pathogen interaction that could complement the gene expression knowledge is still lacking in *P. salmonis*, since a more comprehensive global proteomic analyses can suggest a more direct interpretation of molecular responses in a biological system.

Our study used a global proteomic profiling to identify differentially expressed proteins in macrophage-like cells of Atlantic salmon challenged with *P. salmonis* at different stages of infection. We focused on the host and bacterial processes, rather than individual proteins altered during infection, to discover the defense mechanism of Atlantic salmon against *P. salmonis* invasion, *P. salmonis* induced changes in the host, potential bacterial virulence factors, and their target in host cells.

## 2. Materials and Methods

### 2.1. Bacterial Strains and Culture Conditions

The *P. salmonis* strain LF-89 (ATCC VR-1361) was grown aerobically in nutrient broth (AUSTRAL-SRS [20]) at 16 or 18 °C in a shaking incubator at 140 rpm. Each subculture was confirmed as *P. salmonis* by Gram stain and RFLP [21]. To build the growth curve, bacterial growth was measured every day for 12 days by monitoring the optical density at 600 nm (OD_600_). Bacterial cell numbers were estimated by counting in a Petroff–Hauser chamber, according to manufacturer’s instructions.

### 2.2. SHK-1 Cell Culture Infection Assays

The SHK-1 macrophage-like cell line derived from Atlantic salmon (*Salmo salar*) head kidney (ECACC 97111106), was routinely grown at 20 °C in T25 or T75 tissue culture flasks, with Leibovitz’s L-15 medium (Gibco) supplemented with 2 mM L-glutamine (Gibco), 40 µM β-mercaptoethanol, and 10% FBS (Gibco). For infection assays, cells were seeded at 80% confluency and inoculated with stationary-state bacteria at multiplicity of infection (MOI) ranging from 50:1 to 250:1, as described previously [11]. After three days of co-incubation at 16 °C, gentamicin was added to a final concentration of 50 µg/mL, to kill extracellular bacteria. The antibiotic was incubated for 1 h, washed three times with PBS, and replaced with fresh culture media. The infection assays (in triplicate) were monitored daily under optical inverted microscope (100× magnification) until the appearance of cytopathic effects (CPE).

### 2.3. Global Proteomic Profiling Using Q-Exactive Mass Spectrometry

For proteomic analyses, infected and uninfected (control) SHK-1 cells were processed. Two infection stages were compared: an early-infection stage named vacuolization (6 days post-infection), and a late-infection stage named propagation (12 days post-infection). Planktonic cells grown in broth medium for three days (exponential-state bacteria) were used as *P. salmonis* control condition. Cells from each experimental condition were concentrated by centrifugation at 500× *g* for 10 min and 8000× *g* for 5 min for SHK-1 and bacterial cells, respectively. Sample replicates (*n =* 3 per condition) were pooled together and cell pellets were quickly frozen and kept at −80 °C until further use. Schematic representation of samples obtained for proteome analysis is shown in Figure S1.

Global proteomic profiling of samples representing the different experimental conditions were processed in Bioproximity, LLC (Manassas, VA, USA). Protein denaturation, digestion, and desalting of samples were performed using the filter-assisted sample preparation (FASP) method [22]. Briefly, the samples were digested using trypsin, and each digestion mixture was analyzed by ultra-high-pressure liquid chromatography (UHPLC-MS/MS), coupled to a high resolution, high mass accuracy quadrupole-Orbitrap mass spectrometer (Q-Exactive), Thermo Fisher (Waltham, MA, USA). Mass spectrometer RAW data files were compared with the most recent protein sequence libraries available from UniProtKB. Proteins were required to have one or more unique peptides detected across the analyzed samples with an *e*-value ≤ 0.0001. All proteomics results are listed in Table S1.

### 2.4. Bioinformatics Analysis of Proteins Detected by Global Proteomic Profiling

Data analysis was performed separately for *P. salmonis* and *S. salar*. All known proteins were identified and functionally annotated using the UNIPROT database. For unknown bacterial proteins, functional annotation was performed using NCBI nr database (https://www.ncbi.nlm.nih.gov/), GenDB (https://www.genedb.org/), VFDB (http://www.mgc.ac.cn/VFs/main.htm) [23]) and SMART (Simple Modular Architecture Research Tool). Proteins were classified according to the predicted functions annotated in the Clusters of Orthologous Groups of Proteins (COGs) database (https://www.ncbi.nlm.nih.gov/research/cog) [24]. As *P. salmonis* genome is sequenced and annotated, the UniProtKB ID of each protein was mapped to the EggNOG database of orthologous groups and functional annotation (http://eggnog5.embl.de/) [25]. *S. salar* proteins were identified from Q-proteomics data using: NCBI:txid8030 ID (PubMed Taxonomy database). Proteomes from each experimental condition were compared using an online tool that generates Venn diagrams and lists of proteins detected in any given condition (http://bioinformatics.psb.ugent.be/webtools/Venn/). The proteins detected in two experimental conditions were analyzed by calculating log_2_ values of condition_1/condition_2 detection ratios and expressed as fold-changes.

Enrichment analyses were performed using AgriGO (http://bioinfo.cau.edu.cn/agriGO/analysis.php) [26] for *S. salar* proteins, first with the complete proteome list for each sample (control, vacuolization, or propagation proteome against the reference genome) and afterwards in pairwise comparisons (vacuolization vs. control, propagation vs. control, and propagation vs. vacuolization) considering the proteins significantly up- or downregulated (log_2_ ratio ≥ 0.6 or ≤−0.6). Enrichments analysis for *S. salar* proteins were consider statistically significant when false discovery rate (FDR) < 0.5, *p*-value < 0.01, and log_2_ fold-enrichment > |0.5| (fold-enrichment = percent of enriched proteins in sample/percent of enriched proteins in reference).

### 2.5. Bacterial RNA Purification and Transcripts Quantification

Total RNA was purified from infected and uninfected SHK-1 cultures, as well as from *P. salmonis* batch cultures, as mentioned for the proteomic assay. SHK-1 and *P. salmonis* cells were collected by centrifugation at 8000× *g* for 5 min, washed with sterile PBS, and suspended in RLT buffer (RNeasy ^®^ Mini Kit) (Qiagen, Hilden, Germany). Cells were disaggregated and homogenized with a 27G syringe, and RNA was purified with RNeasy^®^ Mini Kit (Qiagen, Hilden, Germany), as indicated by the manufacturer. Bacterial RNA was purified using the RNeasy^®^ Mini Kit (Qiagen, Hilden, Germany) with the adjusted protocol for bacterial cells. RNA was quantified using a Qubit™ RNA HS Assay kit (Thermo Fischer Scientific, Waltham, MA, USA), and 400 ng of RNA from each sample were used to synthesize cDNA with the High Capacity RNA-to-cDNA kit (Applied Biosystems, Foster City, CA, USA)

Transcripts were quantified using a Takyon qPCR Kit (Eurogentec, Lieja, Belgium) with specific primers (Table S2) designed for selected *P. salmonis* genes that changed its expression during infection in the proteomic assay. Real time quantitative PCR (qPCR) was performed in an AriaMx 1.0 system (Agilent, Santa Clara, CA, USA) with the following PCR conditions: 95 °C for 3 min followed by 95 °C for 3 s, 60 °C for 15 s, and 72 °C for 15 s for 40 cycles. Melting curves (1 °C steps between 60 and 95 °C) ensured that a single product was amplified in each reaction. The geometric median of the housekeeping genes *recF* and *rho* was calculated for each sample, and used to calculate the relative expression levels of the *P. salmonis* genes using the method described by Pfaffl [27]. Results were expressed as average of three independent replicates with the corresponding standard deviation. GraphPad Prism software version 8.0.1 for Windows (GraphPad Software, La Jolla, CA, USA, www.graphpad.com) was used for graphical representation and statistical analysis of the results.

## 3. Results

### 3.1. P. salmonis Infection Progress in Fish Macrophages Culture

**Figure 1:**
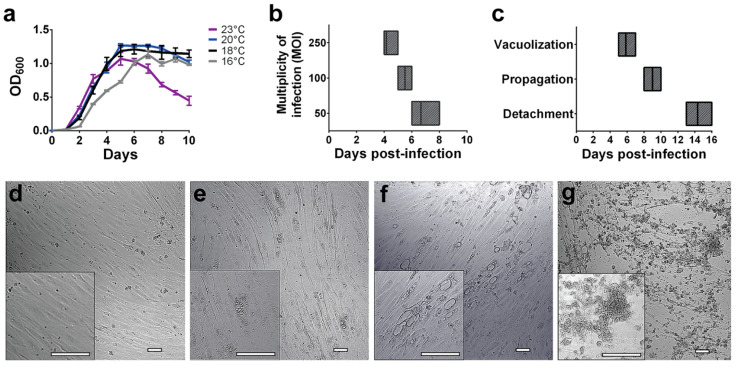
*P. salmonis* growth and infection progression in salmon macrophage-like cells. (**a**) Growth curves *P. salmonis* at different temperatures. (**b**) Day of appearance of CPE in SHK-1 cells using different MOI. (**c**) Day of occurrence of the different infection stages using MOI = 100. Results in (**a**–**c**) are shown as the average of three independent assays with its correspondent standard deviation. (**d**–**g**) Representative bright-field micrographs of *P. salmonis* infected SHK-1 cultures. (**d**) Uninfected SHK-1 cells (control). (**e**) Vacuolization stage, cells showed small and dispersed intracellular vacuoles. (**f**) Propagation stage, characterized by the presence of large vacuoles in groups of cells. (**g**) Detachment stage, where infection disrupted the cell monolayer and lysis of the macrophage-like cells were observed in all fields. Images taken with 100× optical magnification and 300× digital magnification (inset), bar = 100 µm.

To standardize the infection conditions, *P. salmonis* growth was evaluated in liquid culture medium at different temperatures (Figure 1), including the normal temperature of bacterial growth (18 °C), the temperatures used for growth and infection of SHK-1 cells (20 °C and 16 °C respectively), and a higher temperature (23 °C). It was observed that the bacteria grow from 16 to 23 °C with a similar generational time (0.89 days at 16 °C, 0.83 days at 18 °C, 0.99 days at 20 °C, and 0.98 days at 23 °C); however, at 16 °C, the lag phase was extended, and growth at 23 °C increased bacterial decay upon reaching the stationary phase. Between 16 and 20 °C, the OD at 600 nm (OD_600_) remained relatively constant during the stationary phase until 12 days of growth. Therefore, for all the infection assays, bacterial cells from liquid cultures in stationary growth phase at 18 °C were employed. Under these conditions, differences of two days were observed in the appearance of cytopathic effects (CPE, i.e., intracellular vacuoles and distortion of cellular shape) in SHK-1 cells infected with different multiplicity of infection (MOI) (Figure 1b). As the appearance of CPE was similar between MOI 250 and MOI 100, all subsequent infection assays were performed with the lower MOI. The progression of cytopathic effects was evaluated directly under the microscope and three stages of infection were distinguished (Figure 1c–g). The early-stage, vacuolization, was characterized by the appearance of small and disperse intracellular vacuoles (up to three vacuoles per field were observed under a microscope with 100× magnification, Figure 1e). The late-stage, named as propagation, was characterized by the merging of several vacuoles or the increase in vacuolar size, forming large vacuoles throughout the cell culture. Large vacuoles in groups of neighboring cells in all fields were observed (Figure 1f). Finally, detachment was observed as a disruption of the cell monolayer and lysis of the macrophage cells in all the fields under the microscope (Figure 1g). Since a gentamicin treatment was applied to infections after three days of incubation, the remaining bacteria that continued with the infection process were internalized and, therefore, the CPE occurred by the action of intracellular bacteria.

### 3.2. Global Proteomic Profiling of P. salmonis Infection in S. salar SHK-1 Cell Cultures

**Figure 2:**
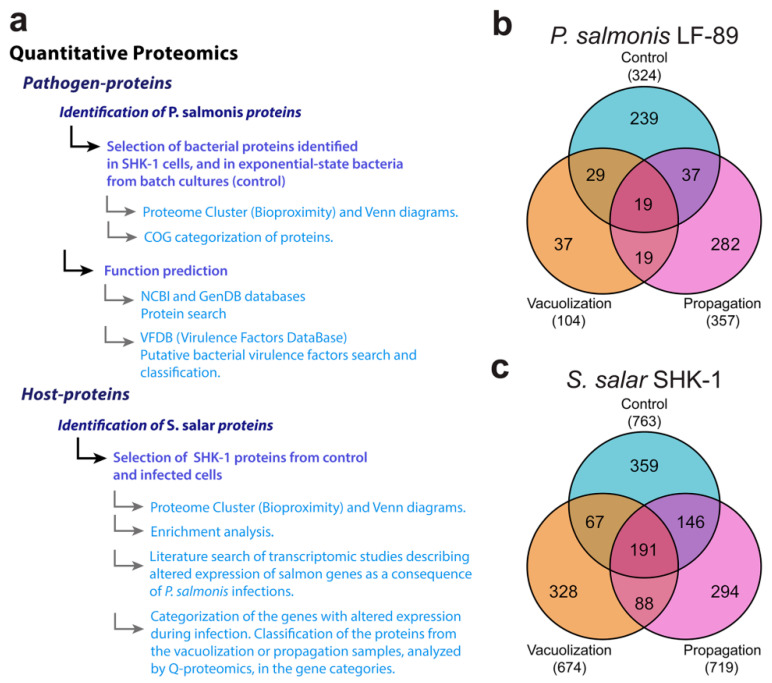
Quantitative proteomics results and identification of proteins. (**a**) Bioinformatics pipeline for global proteomic profiling analysis of *P. salmonis*-SHK-1 infected cells. (**b**,**c**) Venn diagrams of quantitative proteomics results for SHK-1 infections with *P. salmonis*. (**b**) *P. salmonis* proteins identified in control bacteria grown in nutrient broth (exponentially growing bacteria) and intracellular bacteria in SHK-1 at vacuolization and propagation stage of infection. (**c**) *S. salar* identified in control uninfected cells and in different infection stages (vacuolization and propagation).

To compare the expression of *P. salmonis* proteins during the infection process and the effect on the macrophage cells of *S. salar*, SHK-1 infected cells were collected from vacuolization and propagation stages of infection, as well as from uninfected control cells. Additionally, *P. salmonis* cells grown exponentially in a bacteriological culture medium were collected (Figure S1). Global proteomic profiling was performed as previously described [28,29]. As shown in the Venn diagrams (Figure 2b,c), 778 distinct proteins were identified in the vacuolization stage, 674 of them from *S. salar* and 104 from *P. salmonis*. In the propagation stage, 1076 proteins were identified, 719 corresponded to *S. salar* proteins and 357 to *P. salmonis*. Finally, in the control condition of uninfected SHK-1 cells, 763 *S. salar* proteins were identified. Considering that these samples where obtained from equivalent numbers of SHK-1 cells, *S. salar* proteomic profiles from different infection stages could be quantified and compared with the control growth condition. On the other hand, control *P. salmonis* cells were collected from a pure bacteriological culture and the amount of proteins in this condition could not be quantitatively compared with those of bacterial cells obtained from infected macrophages. Additionally, the number of proteins identified in the three conditions (*n =* 19) was low compared to the SHK-1 cell cultures (*n =* 191). Therefore, analysis of the proteomic results of *P. salmonis* was performed differently from the *S. salar* proteomes (Figure 2a).

Each proteome was ordered and categorized according to their ontology. In the case of *P. salmonis*, orphan proteins with unknown function were further analyzed. Sequence similarity searches using the Virulence Factor Database (VFDB) allowed us to identify putative virulence factors in the list of *P. salmonis* proteins with unknown function. A detailed pipeline for the assignment of functions of unknown proteins is depicted in Figure 2a.

#### 3.2.1. *P. salmonis* Proteome

**Figure 3:**
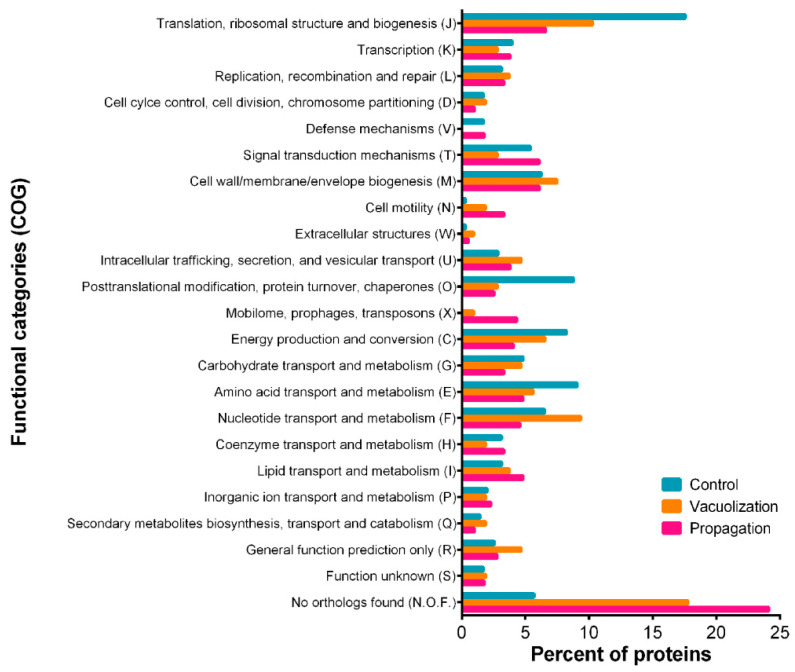
Functional categories of *P. salmonis* proteins Percent of proteins in each COG (Cluster of Orthologous Groups) related to the total bacterial proteins identified by quantitative proteomic for the control condition (exponentially growing bacteria in nutrient broth, blue), vacuolization (bacteria inside SHK-1 cells in vacuolization stage of infection, orange), and propagation (bacteria inside SHK-1 cells in propagation stage of infection, pink).

As shown in Figure 2b, 239 unique proteins were identified during exponential growth of *P. salmonis* cells in nutrient broth, 37 in the vacuolization stage and 282 in the propagation stage of infection. In the bacterial control cells, 324 proteins were identified, a similar number to that obtained in the *P. salmonis* proteomes from the late-infection stage intracellular bacteria, while the lowest number of identified proteins was found in the early stage of infection. In total, 19 proteins were found in all conditions and also 19 proteins were common between the infection stages. This gives a total of 338 bacterial proteins identified exclusively during the infection of salmon macrophages. *P. salmonis* proteins were group in COG categories and quantified in each proteome (Figure 3). Some of the over-represented COGs (75th percentile or above) were common for all conditions, such as translation, structure, and biogenesis of ribosomes (J); cell wall/membrane/envelope biogenesis (M); and amino acid transport and metabolism (E). In a general view, categories related to cellular metabolism, such as energy production and conversion (C), transport and metabolism of amino acids (E), and nucleotides (F) were over-represented in control cells. Particularly, J was the category with the highest number of representatives in the control condition. In addition, post-translational modifications, protein replacement, and chaperones (O) were over-represented.

On the other hand, during the infection, *P. salmonis* proteome showed a high percent of proteins in categories related to cell motility (N), extracellular structures (W), intracellular trafficking, secretion and vesicular transport (U, including secretion system proteins), lipid transport and metabolism (I, including toxins and LPS synthesis and modification proteins), and mobilome prophages and transposons (X). Proteins annotated in the N category were mostly part of the flagellar structure, while pilus and fimbrial proteins were assigned to W. Of note, no transposons or phage-related proteins (X category) were found in control cells. In addition, unknown proteins with no orthologs (N.O.F in the bar chart) represented a high percent of proteins in both bacterial infection stages, but not in control cells. Besides metabolism-related proteins, chromosome partitioning proteins, pilus assembly, and T6SS proteins were present during vacuolization, but not in propagation; in the latter stage, fimbria was represented in W category and T4SS in U. Contrary to the proteome in propagation stage, no proteins in V category (defense mechanisms) were found during vacuolization. Almost 29% of all proteins in propagation stage did not have an assigned function (categories S, R and N.O.F in Figure 3), and the over-represented COGs were J, T, M, E, I, and X. Although T category was in similar proportions in control and propagation stage, in the control condition, this category comprised proteins that regulate nutrient transport and storage, such as carbon and phosphate, while in the propagation stage it was mostly composed by chemotaxis proteins.

#### 3.2.2. *S. salar* SHK-1 Cells Proteome

In total, 2156 host proteins were identified in the proteome data obtained from *S. salar* infected and uninfected SHK-1 cells, and 492 proteins were shared between two or more conditions (Figure 2c). Enrichment analyses for gene ontology (GO) terms related to biological process, molecular function, and cellular component were performed to gain insight into the functionality of host proteins expressed at vacuolization and propagation stages (Table S3).

**Figure 4:**
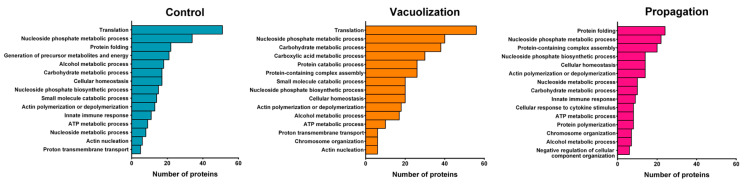
Classification of SHK-1 proteins in most represented Gene ontology (GO) terms. All proteins in control, vacuolization stage, and propagation stage were classified based on GO annotation of SHK-1 proteome, and the 15 most representative biological processes GO terms are shown. The number of proteins in each category for all identified proteins in the three conditions is shown in the bar graph.

Approximately 45% of SHK-1 proteins identified by quantitative proteomics were found exclusively in one condition and, therefore, could not be compared between samples. To explore which biological processes were altered at each stage of infection, enrichment analyses were performed using the total proteomes from each sample. In total, 98, 158, and 107 processes were enriched in the control, vacuolization stage, and propagation stage, respectively. Based on the fold-enrichment and the number of proteins, the 15 most abundant GO terms were ranked, as shown in Figure 4. In a general view, the enriched categories in the vacuolization stage were similar to the control proteome, where the most enriched categories were related to metabolism and energy production. Seven GO terms (nucleoside phosphate metabolic and biosynthetic processes, actin polymerization and depolymerization, alcohol metabolic process, carbohydrate metabolic process, cellular homeostasis, and ATP metabolic process) were over-represented in all proteomes, whereas amino acid metabolism and protein catabolism were over-represented only in the vacuolization sample. Compared to the other samples, the propagation stage showed fewer proteins in each category, and a marked enrichment in categories related to the immune cell response and the regulation of protein complex assembly, protein regulation, and polymerization. It is worth noticing that most of the proteins present in those categories were exclusively found in the propagation stage.

**Figure 5:**
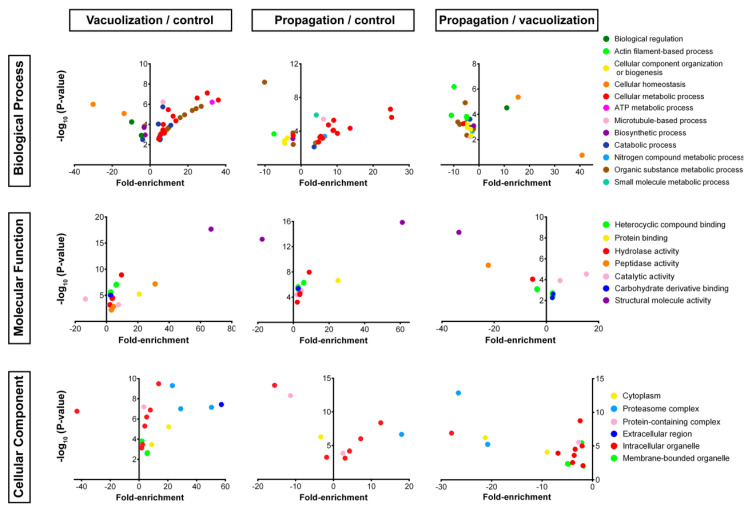
Enrichment analysis of *S. salar* proteins in SHK-1 infected and uninfected cells. Gene ontology (GO) terms were assigned to the proteins identified by quantitative proteomics, and the resulting GO were compared to *S. salar* proteome (reference). Proteins with assigned GO were quantified and each GO is shown as fold-enrichment. For each comparison (vacuolization vs. control, propagation vs. control, and propagation vs. vacuolization), significantly upregulated proteins (log_2_ ratio ≥ 0.6) or downregulated proteins (log_2_ ratio ≤ −0.6) were used for enrichment analysis. Positive values indicate over-represented categories, and negative values under-represented categories for each comparison (indicated with rectangles at the top). Over- or under-represented GO with FDR < 0.05 are shown.

To incorporate the abundance levels of the proteins in each condition, enrichment analysis was performed using only the proteins over- or under-expressed in the infection stages relative to the control uninfected proteome. Considering the biological process, cell compartment, and molecular function categories, 114 significant terms were found among the 119 upregulated proteins in vacuolization stage relative to the control cells, and 35 terms were enriched among the 75 downregulated proteins. Biological process categories highly enriched in the vacuolization stage (Figure 5) were related to energy production and metabolism and included “glycolysis”, “generation of precursor metabolites and energy”, and “cellular amino acid metabolic process”, grouped in the parent GO term “cellular metabolic process” (together with nucleoside, nucleotide, and ribonucleotide metabolic, and biosynthetic processes). Glucose, monosaccharide, hexose, and alcohol catabolic process categories were also enriched, grouped in the parent GO term “organic substance metabolic process” and “ATP metabolic process”. Processes related to protein and amino acids were significantly enriched, as revealed by the terms “amino acid metabolism”, “protein catabolic process”, and “proteolysis” (grouped in the parent GO term “organic substance metabolic process”), together with the molecular function term “peptidase activity”. The most under-represented biological process terms were “gene expression” (parent term “organic substance metabolic process”), and “biosynthetic process”. In addition, processes related to homeostasis were under-represented in vacuolization stage, denoted by the term “homeostatic process” (grouped in the parent term “biological regulation”) and the parent term “cellular homeostasis”, which includes the most under-represented terms in vacuolization, “cell redox homeostasis”. The extracellular region was over-represented in vacuolization stage, mainly due to the enrichment of the “extracellular vesicular exosome” category, a GO unique to this stage of infection. The proteasomal complex and its components, membrane-bounded vesicle, and vesicles were also unique to the vacuolization stage (“membrane-bounded organelle” parent cellular component GO term). When data of the protein abundance were considered, we noticed that enriched processes in Figure 4, such as translation, cellular homeostasis, and catabolic process, were under-represented when compared to uninfected cells.

In the propagation stage proteome, 126 proteins were upregulated relative to the control cells and 60 significant terms were found, whereas 27 terms were enriched among the 118 downregulated proteins. A highly enriched biological process was “cellular metabolic process”, a term comprising diverse nucleoside, nucleotide, nucleobase, and ribonucleotide metabolic and biosynthetic processes (Figure 5), which correlates with the enriched Molecular functions “ribonucleotide binding” (“carbohydrate derivative binding” parent term), “heterocyclic compound binding”, and “nucleoside-phosphatase activity” (parent term “hydrolase activity”). Additional over-represented cellular metabolic processes were organic acid, nitrogen compound, and ketone metabolic processes. As in vacuolization stage, the most under-represented biological process was the parent term “organic substance metabolic process” and specifically “translation”, which correlates with the most under-represented molecular function GO, “structural constituent of ribosome” (parent term “structural molecule activity”). The categories “actin filament-based process” and “biosynthetic process” were also under-represented. Interestingly, “microtubule-based process” was enriched in vacuolization and in propagation stages relative to the control cells, as was the molecular function “structural constituent of cytoskeleton” (“structural molecule activity” parent term), and the cellular component categories of “cytoskeleton”, and specifically “microtubules” (grouped in “intracellular organelle” parent term). As observed above, when the protein abundance levels of propagation stage proteins relative to the control proteome are considered, some enriched terms in Figure 4 corresponds to under-represented GOs in Figure 5, as observed with cellular metabolic process, cellular macromolecular complex assembly and organization, and actin filament-based process.

When comparing the proteome of both infection stages, “homeostatic process” (“biological regulation” parent term) and “cellular homeostasis” were the only enriched biological process categories that increased in propagation over vacuolization, while processes related to actin and microtubule cytoskeleton, gene expression, generation of precursor metabolites and energy, carbohydrate metabolism, and protein catabolism were under-represented in propagation (Figure 5). In the cellular component category, “cytoskeleton”, “microtubules”, “vesicle”, “membrane-bounded vesicles”, “ribosomes”, “protein complex”, and “proteasome complex” were all under-represented in propagation. On the other hand, the molecular functions of “catalytic activity”, nucleotide, ribonucleotide, and ATP binding (grouped in “heterocyclic compound binding” parent term), as well as nucleoside and purine nucleoside binding (“carbohydrate derivative binding” parent term) were over-represented in propagation. Molecular functions terms related to GTP binding (“heterocyclic compound binding” parent term), GTPase activity (“hydrolase activity” parent term), and “peptidase activity” were under-represented in the propagation stage.

Then, we sought to compare individual proteins of interest and their relative expression changes between infected and control cells. As detailed in Figure 2a, previous transcriptomic analyses reporting *P. salmonis* infection in salmon cell lines [30,31] or fish organs [7,8,9,32] were used to identify functional categories of genes differentially expressed in the host during *P. salmonis* infection. *S. salar* proteins associated with these categories were identified in each proteome. A complete list of the categorized proteins, their description, fold-change (when appropriate), and reference (when possible) is provided in Table 1.

Proteins related to iron metabolism were scarce and only found in the infected cell proteomes. Among the proteins related to host immune response during infection with *P. salmonis*, only 20 proteins were found in two or more conditions, and only one of them was increased in the infection condition. Toll-like receptors 5 and 8b1, interleukin receptor 12, interleukin-1 receptor-associated kinase 4, and a leukocyte elastase inhibitor were significantly decreased in the propagation stage compared to the control cells. The pro-inflammatory interleukin 1-beta was identified only in vacuolization, interleukin receptors were found in both infection stages, and two interleukin enhancer-binding factors were increased in the propagation stage when compared to the vacuolization proteome. Interferon-related proteins, as well as the interferon-induced guanylate-binding protein 1 (GBP1), were identified only in the propagation stage, as observed in Figure 4.

Redox and stress response were altered in the infected cells. Heat shock proteins (HSPs), especially HSP90 and HSP70, and the detoxifying proteins glutathione S-transferase A, tryparedoxin, and peroxiredoxin-6 were upregulated in the vacuolization proteome. The same proteins were downregulated in the propagation stage, both relative to the control and the vacuolization stage. In addition, peroxiredoxines PRDX1, PDX5, and TDX were upregulated in propagation stage. Although stress related proteins and radical-detoxifying enzymes were found in the infected cells, enzymes related to the generation of reactive nitrogen species in macrophages (such as nitric oxide synthase NOS2 or iNOS) were not identified in any condition (with the exception of a N-oxide forming monooxigenase significantly increased in vacuolization), while the reactive oxygen species (ROS) generating enzyme NADPH oxidase was found in similar levels in the control and the infected cells during propagation stage.

Proteins related to structure and dynamic of cellular cytoskeleton were differentially expressed in the infected cells. Tubulin and tubulin-related proteins appeared to be upregulated in infected cells, although increased in vacuolization over propagation. Tropomyosin was decreased in vacuolization relative to control cells and propagation stage, myosin proteins were increased in both infection stages, and actin was more abundant in propagation compared to vacuolization stage. Actin-related proteins were found in significantly increased levels, or exclusively in the vacuolization stage.

Finally, proteins related to clathrin-mediated endocytosis, phagosome, and vesicular trafficking were identified in infected samples. Clathrin light chain and clathrin assembly protein PICA were only found in vacuolization stage, and the protease inhibitor cystatin B was increased in this infection stage. Proteins associated with the phagosome (syntaxin-3 and -5), vesicle fusion (synaptotagmin, syntaxins, and syntaxin-binding proteins), endosome (Rab-14 and Rab-18), and LAMP1 (lysosome) were identified in propagation stage.

### 3.3. P. salmonis Virulence Factors

**Figure 6:**
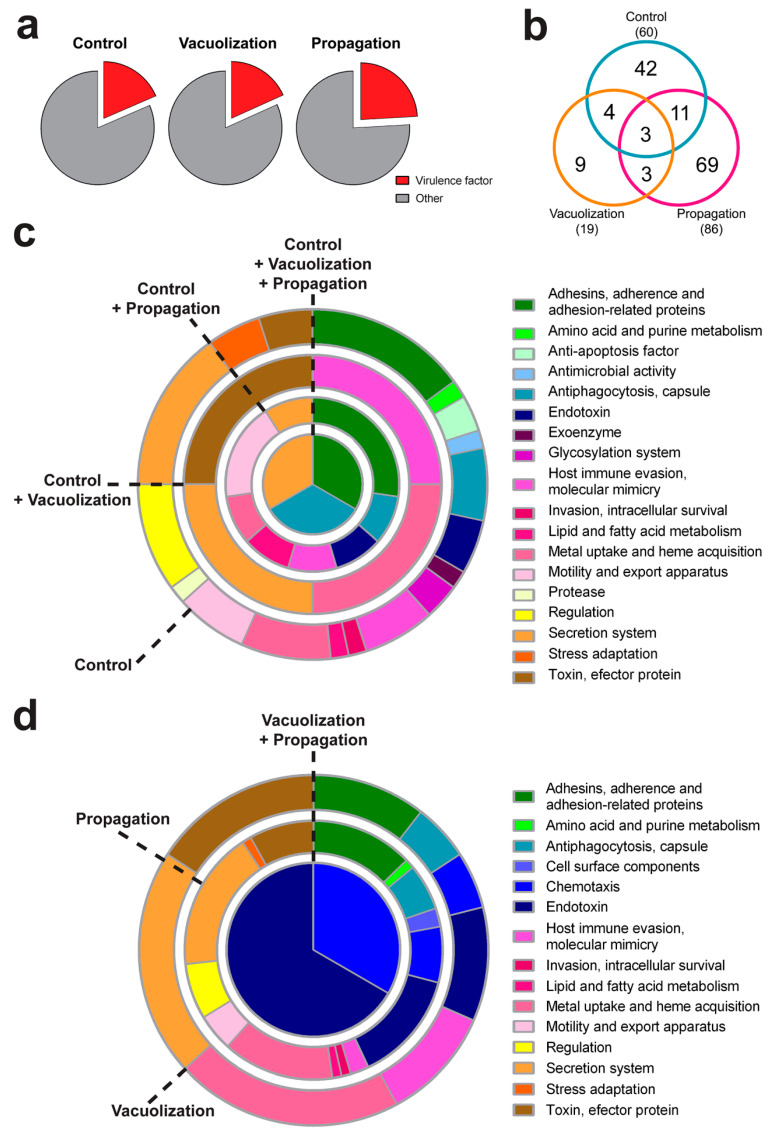
Virulence factors families identified by quantitative proteomics in *P. salmonis* proteomes from control sample, and infection samples in SHK-1 cells (vacuolization and propagation stages of infection). Proteins were classified according to the virulence factor data base (VFDB) family classification. (**a**) Pie charts of the proteins classified as virulence factors (red) in each *P. salmonis* proteome. (**b**) Venn diagrams showing the quantity of virulence factors identified in and shared between the three *P. salmonis* proteomes. (**c**) Virulence factor classification of *P. salmonis* proteins identified in the control, in control and vacuolization, in control and propagation, and in all three samples. (**d**) Virulence factor classification of *P. salmonis* proteins identified only in the infection proteomes.

Bacterial virulence factors were predicted in all proteomes, as described in Figure 2a, and grouped in virulence factor families as classified in the VFDB for other pathogens. About 18% of all proteins in the control and the vacuolization stage, and 24% of propagation-stage proteins, were classified as virulence factors (Figure 6a). Virulence factors were grouped by stage of infection, as shown in Figure 6. Each pie chart represents the percent of virulence factor families that were present in one or more samples. The highest diversity of virulence factor families was observed in the propagation stage. Only three virulence factor families were shared for all three proteomes, and three families were common to the two infection proteomes (Figure 6b).

“Adhesins, adherence and adhesion-related proteins”, “endotoxin”, “host immune evasion, molecular mimicry”, “metal uptake and heme acquisition”, and “secretion system” were the more abundant families, summing up a total of 50%, 74%, and 60% of the virulence factor proteins in the control, vacuolization stage, and propagation stage, respectively (Figure 6c). “Anti-apoptosis factor”, “antimicrobial activity”, “exoenzyme”, “glycosylaton system”, and “protease” were the virulence factor families found exclusively in the control cells. In addition, “amino acid and purine metabolism”, “lipid and fatty acid metabolism”, “invasion, intracellular survival”, “motility and export apparatus”, “regulation”, and “stress adaptation” families were common for the control and propagation stage conditions (Figure 6c). There were no common families between the control and the vacuolization stage bacterial proteome.

Although some virulence factor families were present in all samples, the identity of those virulence factors differed. For example, the highly represented “secretion system” family was composed mainly by T6SS proteins in the control and in the vacuolization stage proteome, but in the propagation stage the secretion system proteins belonged to the Dot/Icm T4SS. In addition, in “adhesins, adherence and adhesion-related proteins” family, there were common proteins to all conditions, with the exception of flagella-related proteins, which were found exclusively in intracellular bacteria in both infection stages.

On the other hand, “cell surface component” and “chemotaxis” families were exclusive for intracellular bacteria inside the infected cells (Figure 6d). Although no families were found to be exclusive for the vacuolization-stage bacteria, “amino acid and purine metabolism”, “cell surface components”, “invasion, intracellular survival”, “lipid and fatty acid metabolism”, “motility and export apparatus”, “regulation”, and “stress adaptation” families were found exclusively in the propagation stage (Figure 6d). The virulence factor family “cell surface components” was also exclusively found in propagation stage bacterial proteome.

### 3.4. Gene Expression of Selected P. salmonis Virulence Factors

**Figure 7:**
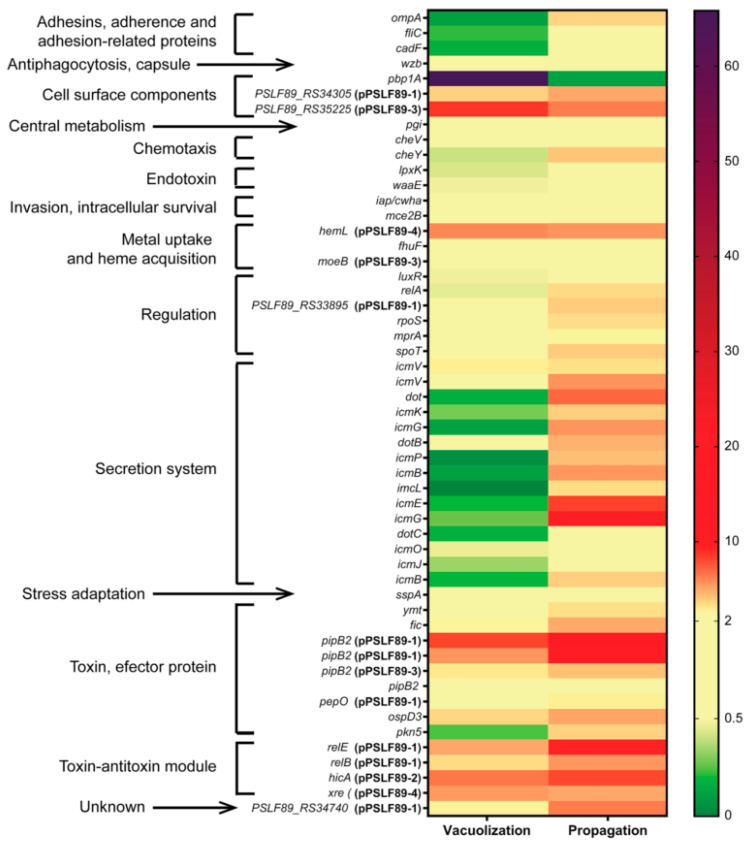
Heat map showing expression levels of *P. salmonis* virulence factors in SHK-1 infected cells. Expression levels of *P. salmonis* genes are expressed as ΔΔCt values normalized by exponential culture bacteria as control, and *recF* and *rho* genes as housekeeping genes. ΔΔCt values were used to create a heat map: values between 0.5 and 2 (yellow) correspond to expression levels similar to control bacteria, increased expression relative to control bacteria is shown in red and purple, and decreased expression in green. Virulence factors were classified in families as before (Figure 5).

To validate the differentially expressed proteins at a transcriptional level, and to evaluate the expression of selected virulence factors families of the infected proteomes, qPCR analysis was performed. The genes selected for evaluation belonged to virulence factor families relevant to the infective process, as shown in Figure 7. Most of these genes were identified in our proteomic data, but also others were evaluated because of their proximity to the identified genes or forming a gene cluster (*dot/icm* genes and toxin-antitoxin modules), or they were present in more than one copy (*pipb2* genes). All evaluated *P. salmonis* genes, their location (chromosome or plasmid), the virulence factor prediction and family, the proteome sample (if any), and the ΔΔ*C*t values are listed in Table S4. Overall, 12 of the 54 selected genes did not change their expression during infection compared to the exponentially growing bacterial cells (control). A “central metabolism” family gene (*pgi*), a “stress adaptation” gene (*sspA*), and proteins related to membrane, cell wall, and surface components (*wzb*, *waaE*, *iap/cwha,* and *mce2B*) showed similar relative expression levels in control and intracellular bacteria from infected cells. The stationary phase-related genes *rpoS*, *relA*, and *spoT* were upregulated only in the late infection stage (propagation). In the vacuolization stage, 19 genes were downregulated, 13 were upregulated, and 22 were similar to the control condition. All the genes that belonged to the “adhesins, adherence and adhesion-related proteins”, 2 out of 7 genes from the “regulation”, and 11 out of 15 from the “secretion system” virulence factor families were downregulated. In the propagation stage, only one gene was downregulated, 36 were upregulated, and 17 were similar to the control condition. Most genes from “secretion system” (12 out of 15) and 9 out of 10 genes from “toxin, effector protein” families were upregulated in the propagation stage bacteria. The evaluated T4SS genes corresponded to two of the three Dot/Icm gene clusters found in *P. salmonis* chromosome [11]. These gene clusters showed different behavior in the vacuolization and propagation stages, as most of them were upregulated in propagation, while being downregulated or unchanged in the vacuolization stage.

Additionally, 47% of all genes upregulated during infection were plasmid-encoded. In the global proteomic profiles, 3.1% of the identified proteins in the control sample were encoded in a plasmid, while the same occurred for 4.5% and 6.7% of the vacuolization and propagation proteomes, respectively. Considering that about 10.2% of *P. salmonis* proteins are plasmidial, and that plasmid proteins found in the infected cells appeared to be twice as many as the control in the proteomes, we include them in the qPCR analyses. It is interesting to note that 6 out of 10 tested genes encoded in pPSLF89-1 plasmid were upregulated in vacuolization, while all 10 were upregulated in propagation (Figure 7). Of the unchanged pPSLF89-1 genes in vacuolization, two of them were toxins, one was a surface component and the other a regulator. Regarding the toxins, both pPSLF89-1 copies of *pipB2*, and the pPSLF89-3 copy were upregulated in both stages of infection (Figure 7), although the chromosomal copy of *pipB2* did not change its expression relative to the control. The only plasmidial gene that did not change its expression in both infection stages was *moeB* in pPSLF89-3, while all the tested toxin–antitoxin genes were upregulated in both stages of infection.

## 4. Discussion

*P. salmonis* is a fish intracellular pathogen that causes severe salmonid mortality with costly economic and social impacts in aquaculture. This situation has encouraged research and development of novel therapeutics toward this pathogen. Considering the importance of understanding host–pathogen interactions during *P. salmonis* infection, the present investigation aimed to unravel the cellular response of Atlantic salmon against *P. salmonis* infection and those virulence factors that contribute to bacterial pathogenesis by using global proteomic profiling of infected macrophages. In addition, we further characterized *P. salmonis* macrophage infection at different stages to discover novel biomarkers of early- and late-infection stages.

A standardized infection method was developed based on previous work [11], which allowed us to discriminate between two infection stages: an early stage (vacuolization) characterized by the appearance of small focalized intracellular vacuoles in the SHK-1 cells and a late stage (propagation) when the large intracellular vacuoles spread across the cellular monolayer. Since the first stage occurred shortly after the bacterial infection and the second stage was closer to the cellular rupture (and consequent bacterial release), we sought to further investigate the host–bacteria interaction in these different infection stages, as deciphering *P. salmonis* virulence factors inside host cells can provide a better understanding of the virulence traits of this fish pathogen.

**Figure 8:**
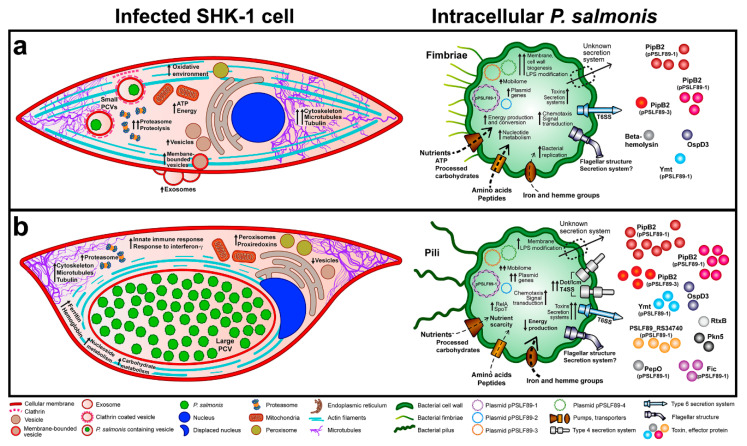
Schematic model of host–pathogen interaction during *P. salmonis* LF89 infection in salmon macrophage-like cell line. The infection model is constructed based in this work and in previous results [7,9,11,14,31,32,33]: the early infection stage (vacuolization) (**a**); and the late-infection stage (propagation) (**b**). (**a**) Bacteria are internalized by clathrin-dependent endocytosis. Inside the SHK-1 cell, *P. salmonis* highjacks the cellular metabolism, increasing energy and ATP production via glycolysis and sugar metabolism. Carbohydrates, amino acids, peptides, iron, and other nutrient transport is increased in the bacteria inside the small PCVs. The host-cell shape begins to be altered and components of vesicular systems increase. Biological quality regulation, gene expression, and cell redox homeostasis decay in SHK-1 cells, forming an oxidative environment. The bacteria respond by producing catalase, thioredoxin and chaperones. The bacteria dysregulate the host cytoskeleton, altering the actin filaments and microtubules, probably by the secretion of chromosome- and plasmid-encoded toxins. Bacteria inhibit their detection by interfering with the host cell immune response by an unknown mechanism related to toxins and T6SS effectors. (**b**) When the infection thrust forward, the size and number of intracellular vesicles increase along with the number of intracellular bacteria. As *P. salmonis* replicates inside large cytoplasmic vacuoles, the nutrient transport to the bacterial cytoplasm decrease, and the stringent response is triggered. Iron becomes limited, different iron acquisition proteins increase in *P. salmonis*, while iron-transporting proteins are found in SHK-1 cells. General nucleotide, nucleoside, and ribonucleotide metabolic and biosynthetic processes increase in both *P. salmonis* and host cells. Propagation stage host cells detects the bacterial pathogen and elicits an immune response via interferon-γ. The expression of Dot/Icm T4SS genes, toxins, and effector proteins increases in *P. salmonis*, together with plasmid genes, mobilome, transposons, and phage-related proteins. The increase expression of secretion system proteins, toxins, and effector proteins suggests the bacteria is preparing to exit the cell (by a yet unknown mechanism) moving forward to the detachment stage of infection, as depicted in Figure 1g.

Based on microarray analysis, Rise et al. proposed a set of molecular biomarkers of *P. salmonis* infection in Atlantic salmon macrophages [8]. They suggested a panel of nineteen host genes that responded with differential expression during infection with the bacteria. In our work, only seven proteins corresponding to the gene biomarkers were identified in the *S. salar* proteomes. Of those seven proteins, four were found in infected cells: CLIC4, with similar levels in infected and control cells; glutathione S-transferase P and A, which were upregulated in vacuolization; and ependymin, found only in vacuolization. Thus, our proteomic results do not correlate completely with those found by microarray analysis, which highlights the different outcomes that can be obtained when comparing the proteins with gene expression. As a result, we propose to observe alterations in biological processes, such as ROS production with concomitant increase in HSPs and peroxiredoxins, actin cytoskeleton or microtubule organization, energy conversion, and ATP production as indicatives of *P. salmonis* infection in *S. salar* cells. Considering those processes, a model of *P. salmonis* infection is suggested based on our global proteomic results, including novel and previous findings using different omics strategies (Figure 8).

Previously, clathrin-dependent endocytosis has been implicated in the internalization of *P. salmonis* in macrophage cells [32,33]. Proteins related to this process, such as clathrin light chain and clathrin assembly protein PICA were identified in the SHK-1 cells only in the vacuolization stage (Table 1). Cell wall/membrane/envelope biogenesis was a predominant category in vacuolization over control bacteria. In this stage, proteins belonging to the “endotoxin” virulence factor family were also abundant, suggesting the bacteria modifies or overproduce surface components, including the LPS, allowing its survival inside the host (Figure 8a). This has been previously described for other intracellular pathogens, such as *Francisella tularensis*, which modulate the LPS and other surface components in order to survive and replicate inside macrophages [34].

An important difference between the vacuolization and the propagation infection stages is related to the cell metabolism and energy production. In the SHK-1 cells infected in vacuolization stage, the biological processes “glycolysis”, “generation of precursor metabolites and energy”, “ATP metabolic process”, and “cellular amino acid metabolic process” were highly over-represented (Figure 4 and Table S3). In addition, protein metabolism was shifted, as the bacteria increased the amino acid metabolism, proteolysis and proteasomal protein catabolic process. The proteasomal complex and molecular functions related to protein degradation were also over-represented in the vacuolization stage SHK-1 cells (Figure 4 and Table S3), reflecting the bacterial nutritional requirements to grow inside the host cells. Bacterial alteration of biosynthesis and degradation of amino acids and proteins has also been reported in transcriptomic studies [14], as well as the bacterial nutritional requirements due to the lack of amino acid-synthesis pathways [20,35]. Nucleotide transport and metabolism were over-represented in both the bacteria and the host cells and can also be contributing to bacterial growth as nutrient or energy source. Here, we propose that *P. salmonis* highjacks the host cellular metabolism in order to obtain the nutrients required for its intracellular replication (Figure 8a). As the bacteria prepares the cell to sustain its replication, host gene expression decay, as well as processes related to the regulation of biological quality, such as protein folding, cellular homeostasis, and cell redox homeostasis (Figure 8a).

Regarding the early cellular response to *P. salmonis*, ROS- and N-oxide-forming proteins were identified in infected SHK-1 cells (Table 1), which is in agreement with the respiratory burst response to pathogen infection in fish [36], although the enzyme NADPH-oxidase (NOX2), which is pivotal to the respiratory burst, was not found at any infection stage. Along with the oxidative environment, HSP70 and HSP90 were upregulated in the SHK-1 cells during the early stage of infection (Table 1). HSPs have an immune regulatory function during infection in cytoprotection against tissue damage associated with inflammation [37,38]. Previously, the upregulation of HSP90 and HSP40 in three tissues (liver, head kidney, and muscle) and in macrophages of Atlantic salmon has been reported [7,8], which is in accordance with our results and reflects the importance of these chaperone proteins during *P. salmonis* infection. In addition, HSP70 has been linked to lysosomal stabilization after permeabilization takes place by ROS and inflammation in response to intracellular pathogens [39]. The “membrane-bound vesicles”, “vesicles”, and “extracellular vesicular exosome” terms were over-represented in the vacuolization, but not in the propagation stage (Figure 4). Although vesicular trafficking and extracellular exosomes have multiple functions in the cells, it has been suggested that they play an important role in the release of cytokines [40] and HSPs (especially HSP70 and HSP90) [38] during the inflammation process. As a result, the bacteria express catalase, thioredoxin, and chaperones to counteract the oxidative environment (Table S1 and Figure 8a). This, together with a decrease in other host cellular processes, such as the regulation of cellular homeostasis, ribosomes, and translation process, suggests the bacteria modify the host cell gene expression and alter the host REDOX homeostasis, as a result of the host immune response to the pathogen challenge (Figure 8a).

In addition, proteins associated with the anti-oxidative response were upregulated in the vacuolization stage, such as glutathione S-transferase P, GTSA, PDX6, and tryparedoxin (Table 1). In response to pathogen infection, peroxiredoxins gene expression varies depending on the tissue or cell type, the fish species, and the stimulus [41,42]. *P. salmonis* seems to be specifically altering some peroxiredoxins as part of its infection strategy, since PDX4 levels were similar in infected and uninfected cells, while PDX6 was upregulated in was upregulated in vacuolization, and TDX, PDX1, and mitochondrial PDX5 were upregulated in the infected SHK-1 cells during the propagation stage (Table 1).

Although a ROS-forming environment appears to be elicited in infected cells, neither Nf-kappa-B nor Nf-kappa-B-induced proteins were identified during *P. salmonis* infection in vacuolization stage. In addition, the canonical ROS response that activate immune genes and the inflammatory response is absent (Table S1), suggesting the bacteria modulate or interrupt the downstream ROS response that leads to cellular inflammation and, eventually, cell death. Thus, by a still unknown mechanism, the host cell innate immunity is not activated in the early-infection state. Chemotaxis, endotoxin (LPS) alteration, flagellar and fimbriae proteins, T6SS, and the toxins beta-hemolysin and Ymt are some of the *P. salmonis* virulence mechanisms that were present in the vacuolization stage bacteria and can be related to the host immune response adjustments (Figure 8a).

As the infection progress, the host-cell shape begins to transform internally and externally. The extracellular vesicular exosome term was increased in vacuolization stage, along with membrane-bounded vesicles and vesicles in general (Figure 8b). In addition, the bacteria dysregulate the actin cytoskeleton by the differential expression of actin-related proteins (ARPs) and actin-capping proteins. Although not every type of actin protein was increased in the infection stages compared to the control cells, most of them were more expressed in the propagation relative to the vacuolization stage (Table 1). Specifically, our findings suggest that the reorganization of actin cytoskeleton in actin stress fibers, which has been shown in fluorescence microscopy imaging [11,33,43], could be related to changes in ARPs as a result of *P. salmonis* infection (Figure 8). This is in agreement with previous observations reporting that a reorganization of the actin cytoskeleton during infection with *P. salmonis* allows the formation of *P. salmonis*-containing vacuole (PCV) (Figure 8). In addition, it has been suggested that *P. salmonis* induces the synthesis of actin molecules during infection [33]. Although in this work we did not explore these results further, there is extensive literature that relates actin cytoskeleton dynamics to the *P. salmonis* infection in salmon cells [7,11,12,31,32,33,43,44,45]. Interestingly, not only the actin cytoskeleton was affected by *P. salmonis* infection. The GO terms “microtubules” and “microtubule-based process” were over-represented in both infection stages, and tubulin proteins were increased in the infection stages relative to control cells (Figure 4 and Table 1). The modulation of microtubules dynamics is a common mechanism for intracellular pathogen survival [46,47,48]. However, to our knowledge, microtubule alteration has not been described as a mechanism related to *P. salmonis* infection.

As *P. salmonis* replicates inside large cytoplasmic vacuoles, it faces nutrient scarcity (Figure 8b). The propagation stage host cells in did not exhibit the increase in ATP, amino acids, and overall energy production observed in vacuolization. This is also supported by the observation that the bacteria inside the propagation stage cells showed stationary-state bacteria attributes, as seen by the increased expression of stationary phase sigma factor *rpoS*, and stringent response genes *relA* and *spoT* (Figure 6). In addition, the nitrogen-starvation RNA polymerase sigma-54 factor (RpoN) was found in the propagation stage bacteria (Table S1). In other pathogens, this alternative sigma factor has been shown to positively regulate virulence factor expression, motility, quorum sensing, and biofilm production [49,50,51,52]. In *P. salmonis*, flagellar structures, fimbrial proteins, toxins, and effectors were found in the propagation stage. Components of two gene clusters of Dot/Icm T4SS were upregulated, in addition to chromosomal and plasmid-encoded toxins. The majority of virulence factor proteins were identified in the propagation stage, representing over 24% of the identified proteins in this stage (Figure 6a,b). The most represented virulence factor families in the bacteria propagation stage proteome were “secretion system”, “metal uptake and heme acquisition”, “endotoxin”, and “adhesins, adherence and adhesion-related proteins”. Similar results were obtained for the bacteria during vacuolization stage, but the identity of the proteins in each family differed between both infection stages. For example, we observed proteins related to the T6SS in the vacuolization stage, and Dot/Icm T4SS in the propagation stage. These results, in addition to the upregulation of two *dot/icm* gene clusters in propagation stage bacteria (Figure 6), suggest that this secretion system activates in a later infection state, when it is probably required to continue to infect other cells [11]. Previously, it has been proposed that *P. salmonis* could modulate the cellular immune response by secreting effectors from the Dot/Icm T4SS, allowing its intracellular replication [31,53], however, the role of the T6SS in *P. salmonis* virulence has not been explored. Proteins such as the outer membrane fibronectin-binding protein, fimbrial proteins, and flagellar structures were identified in the propagation stage bacteria, as part of the “adhesins, adherence and adhesion-related proteins” virulence factor family. The over-representation of proteins related to the flagellum and pilus cell structures was unexpected since *P. salmonis* has been described as a non-motile bacteria [54,55]. These proteins were also part of the COG categories cell motility (N) and extracellular structures (W), which were over-represented in the intracellular bacterial proteomes. Both the fimbriae and the bacterial pilus can participate in adherence to substrate, such as host tissues, or in DNA transfer, as in the case of the conjugative pili [56]. It is interesting to note that fimbrial proteins were found exclusively in the propagation stage of infection, while pili proteins were found in the vacuolization stage bacteria. Flagellar proteins were identified both in vacuolization and in propagation stage bacteria, although flagellin was found only in propagation. In agreement with our proteomic findings, flagellar genes have been reported to express inside host cells during *P. salmonis* infections, suggesting an alternative role to flagellar structures during intracellular growth [13]. In this regard, in pathogenic bacteria, flagella have also been described as another secretion system [57].

Propagation stage host cells have recognized the pathogen presence and an innate immune response has been elicited, mainly by the cellular response to interferon-gamma (Figure 4). The inflammation response, however, did not trigger cell death as seen by the maintenance of the cellular monolayer integrity (Figure 1f), and the absence of key factors in cell death pathways (as caspases and apoptosis-inducing factors), and pore-forming proteins (gasdermin E, perforins, pannexins, or granzymes, among others). The infected SHK-1 cells in the propagation stage exhibited increased nucleoside-phosphatase activity and nucleoside, nucleotide, nucleobase, and ribonucleotide metabolic and biosynthetic processes (Figure 5). On the other hand, proteins related to nucleotide transport and metabolism were identified in the bacteria, such as nucleosidases, nucleotide hydrolases, ribonucleoside triphosphate deaminase, and ribonucleoside-diphosphate reductase (Table S3). Although this suggests an interconnection between the host and the bacteria nucleotide metabolism, the function in the bacterial growth or infection process is unknown.

Several peroxisome-related proteins (such as peroxisome proliferator-activated receptor beta2B, peroxisomal proliferator-activated receptor A-interacting complex, peroxisomal 2,4-dienoyl-CoA reductase, peroxisomal 3,2-trans-enoyl-CoA isomerase, peroxisomal membrane protein PEX13, peroxisomal coenzyme A diphosphatase NUDT7, and Lon protease homolog 2, as listed in Table S1) were found exclusively or increased in propagation, thus suggesting *P. salmonis* could be specifically altering this organelle as part of its infection strategy. Accumulating evidence report vital role for peroxisomes in the maintenance of cellular redox equilibrium in eukaryotic cells, and have been identified as pivotal regulators of immune functions and inflammation during infection [58,59]. To our knowledge, no interaction between *P. salmonis* and host peroxisomes and peroxiredoxins have been reported in salmon infected cells. However, peroxisomes have been pinpointed as key innate immune effectors to resolve microbial infection in the *Drosophila melanogaster* and murine macrophages infected with *Staphylococcus aureus* and *Escherichia coli* [58].

When the infection thrust forward, the size and amount of intracellular vesicles increases (as observed in Figure 1e–f), along with the number of intracellular bacteria [11]. This is accompanied by an increase in microtubule-based process, tubulin, and actin proteins (Figure 8b). Nevertheless, the decrease in translation and ribosomes in this stage suggests that these changes are initiated and set by the early-infection stage. Phagosome- and lysosome-related proteins were also detected in this stage. In addition to Dot/IcmT4SS, bacteria in the propagation stage increased the expression of toxins and effector proteins. (Figure 6), such as phospholipases, which may participate in bacterial scape from the intracellular compartments, and PipB2, which has been shown to modify the endosome/lysosome distribution along microtubules via the filament extension to elicit bacterial replication inside *Salmonella*-induced filaments (Sifs [60]). These results suggest that during the propagation stage bacteria contain the necessary elements and signaling systems activated to exit the host cells.

In the host proteomic study, we found proteins associated with iron acquisition (ferritin and hemoglobin HBA) and iron-responsive element (IREB2) in the propagation stage. Consequently, in *P. salmonis* proteome in the propagation stage, different iron acquisition proteins were detected, including heme and siderophore receptors, heme response regulators, ferric and ferrous iron transporters, and ferrichrome transporters (Figure 8b). The bacterial requirement for iron has been previously studied [9,13], and restriction of iron availability has been described as a central mechanism to resist *P. salmonis* infection in salmon cells [9,32]. These results are in compliance with previous *P. salmonis* transcriptomic assays, in which the *feo* system and siderophore genes were upregulated inside infected cell cultures [13]. “Metal uptake and heme acquisition” was one of the most represented virulence factor families in the intracellular bacteria (Figure 6), a category that grouped two principal iron-acquisition systems: heme synthesis and transport and siderophore and iron transporters. Intracellular bacteria in vacuolization and propagation stages expressed proteins from both iron-acquisition systems, but with different identities: pyoverdine synthesis protein PvdJ was identified in vacuolization bacteria, while pyochelin proteins PchA and PchH were found in propagation (Table S1). Whether this difference in proteins translates in differential siderophore production remains to be elucidated.

Interestingly, proteins from mobilome, prophages, and transposons were found in higher proportions in the intracellular bacteria (Figure 3 and Table S1). No proteins related to gene transfer or prophage categories were identified during the exponential growth in control bacterial cells. In addition, plasmid related genes were upregulated in the intracellular bacteria, and specifically in the propagation stage (Figure 6). This strongly suggests a role for the mobilome or phage-derived proteins during *P. salmonis* infection. In addition, many virulence factors upregulated in both infection stages corresponded to plasmid-encoded proteins (Figure 6), which supports the hypothesis of the importance of *P. salmonis* plasmids in the infective process [61].

Finally, it should be noted that categories related to unknown proteins—predicted function only (R), unknown function (S), and proteins with no associated category—represented the higher percentage of the proteins identified during macrophage infection. This was in compliance with other studies, as an estimated over 20% of bacterial proteins do not have an assigned function, and that percentage could be higher in pathogenic bacteria [62,63,64]. However, given the high number of identified proteins in *P. salmonis* that have not been previously characterized and/or have unknown functions, many topics remain to be answered and, therefore, further analysis are needed to investigate the role of this unknown proteins in *P. salmonis* pathogenesis.

## Figures and Tables

**Table 1:** List of *S. salar* proteins identified by quantitative proteomics and classified in protein categories. SHK-1 proteins were identified (UNIPROT id) and quantified in uninfected cells (control sample), SHK-1 cells infected with *P. salmonis* in vacuolization stage, and in propagation stage. Significantly upregulated proteins (log_2_ ratio ≥ 0.6) are colored red, downregulated proteins (log_2_ ratio ≤ −0.6) in green, and unchanged in gray. “C” indicates the protein was found only in control sample, “V” only in vacuolization sample, and “P” only in propagation sample. The reference column indicates if a protein was found and reported previously.

UNIPROT ID	Description	Fold-Change Vacuolization vs. Control	Fold-Change Propagation vs. Control	Fold-Change Propagation vs. Vacuolization	Reference
Iron metabolism					
B5XCL8	Lipocalin	V	P	−0.785	[14]
B5X740	Ferritin		P	P	[9,32]
C0H8L6	Hemoglobin subunit alpha HBA		P	P	[12]
C0PU56	Iron-responsive element-binding protein 2 IREB2		P	P	
Immune response					
B5X1Q8	Leukocyte elastase inhibitor ILEU	−0.388	−0.700	−0.312	
Q2QEY8	MHC class I alpha 1 antigen	−4.54	C	V	
B9EPF1	Proteasome activator complex subunit 1 PSME1	−3.26	C	V	
B5X3L4	B-cell lymphoma 6 protein homolog BCL6	C	−0.994	P	
B9ENT4	CCAAT/enhancer-binding protein delta	C	0.580	P	[7]
B9EQ10	GTPase IMAP family member 7 GIMA7	C	−0.0038	P	
T2L2I0	Interleukin-1 receptor-associated kinase 4	C	−2.05	P	
C0H8Y1	Interleukin-12 receptor beta-2 chain	C	−1.36	P	
C0H9Y8	Interleukin-6 receptor subunit alpha	C	−0.321	P	
F5B7Y2	Membrane toll-like receptor 5 TLR5	C	−0.321	P	[12,30]
Q8HX42	MHC class I	C	0.861	P	[9]
B5X3J8	Mothers against decapentaplegic homolog SMAD4	C	−2.23	P	
F5B7Y3	Soluble toll-like receptor 5 TLR5	C	−1.02	P	[12,30]
B5X7I0	TNF receptor-associated factor 5	C	−1.66	P	
S0F1A6	Toll-like receptor 8b1	C	−2.05	P	
B5X6C0	Gamma-interferon-inducible lysosomal thiol reductase GILT	V	P	−2.42	[12]
B5X1U2	Interleukin enhancer-binding factor 2 homolog ILF2	V	P	4.81	
B5X2T5	Interleukin enhancer-binding factor 3 homolog ILF3	V	P	2.75	
C0PUM0	CD166 antigen homolog	V		V	
B5X3Y9	CD99 antigen	V		V	
B5XAB6	Complement component 1 Q subcomponent-binding protein, mitochondrial	V		V	
B5XFB9	Complement factor D	V		V	
Q6IWH5	Interleukin-1 beta	V		V	
C0H963	Interleukin-17 receptor A	V		V	
C0HAA1	Interleukin-31 receptor A	V		V	
B9ELT2	Lysozyme g	V		V	
D0UGE1	MHC class I antigen Sasa-ZBA	V		V	
B5XAL9	C-C motif chemokine 13 CCL13		P	P	[7]
B9ENV6	CD40 ligand		P	P	
A4ZHU6	CD4-like protein		P	P	
Q5ILA0	CD8 beta splice variant		P	P	[7]
C0HBS7	Complement C1q subcomponent subunit B		P	P	[7]
B5X7J5	Complement C1q-like protein 2		P	P	
C0H9W8	Cytokine receptor-like factor 3		P	P	
X2JBZ1	CRFB1b. Type I IFN receptor		P	P	
B5X149	Interferon regulatory factor 2-binding protein 2-A		P	P	
B5X3F0	Interferon regulatory factor 2-binding protein 2-B		P	P	
B5X2U0	Interferon-stimulated 20 kDa exonuclease-like 1		P	P	
B5XA65	Lysozyme		P	P	[9]
B9EP53	Metalloendopeptidase HCE2		P	P	
C0H9L0	Metalloendopeptidase BMP1		P	P	
C0H9S3	Neutrophil cytosol factor 2		P	P	
B5X678	NF-kappa-B inhibitor-interacting Ras-like protein 2		P	P	
V9Q6E2	Suppressor of cytokine signaling 2a		P	P	
B9EP15	T-cell surface antigen CD2		P	P	
A0A0F6QNL8	Toll-like receptor 3		P	P	
B5XCC4	Tumor necrosis factor receptor superfamily member 11B		P	P	
ROS and stress response					
B5DG64	Heat shock protein 90kDa alpha (Cytosolic) class B member 1	1.64	2.19	0.546	
C0HAB6	Heat shock protein 90-alpha 1	1.06	1.48	0.417	
U5KQM7	Heat shock protein 90-alpha 2	0.883	1.48	0.598	[7,8,9]
Q9W6K6	Heat shock protein 90-beta 1	−0.151	1.08	1.23	[7,8,9]
B9EMS3	Heat shock protein 90-beta	2.89	0.733	−2.16	[7,8,9]
C0HBF1	60 kDa heat shock protein CH60, mitochondrial	2.25	−2.91	−5.16	
B5DG30	Heat shock protein 70 isoform 3	0.978	−0.688	−1.66	
B5DFX7	Heat shock cognate 70 kDa protein	0.187	−0.906	−1.09	
B5X3U6	Heat shock cognate 70 kDa protein HSP70	−0.0341	−0.778	−0.744	
B9ENS1	Glutathione S-transferase A GSTA	2.01	−1.04	−3.06	
B5X9L4	Peroxiredoxin-4 PRDX4	0.534	0.421	−0.116	
C0HBF4	Tryparedoxin	3.31	−0.503	−3.81	
B5X5Q6	Peroxiredoxin-5 PRDX5, mitochondrial	−1.94	0.623	2.56	
B5X7X7	Peroxiredoxin-1 PRDX1	−1.19	1.43	2.62	
B5X8H5	Peroxiredoxin TDX	−0.549	0.524	1.07	
B5X9Q1	Peroxiredoxin-6 PRDX6	2.96	C	V	
B5X0U9	Dimethylaniline monooxygenase [N-oxide-forming]	3.20	C	V	
C0PUG5	Heat shock 70 kDa protein 4	1.34	C	V	
Q0KFS6	Heat shock 90kDa protein	C	0.777	P	[7,12]
B8YQA0	NADPH oxidase 1	C	0.179	P	
B5XD88	S-adenosylmethionine synthetase METK2	C	−3.42	P	[12]
Q8UWF4	Heat shock protein 60	C	−4.33	P	
B5DG46	Stress-induced-phosphoprotein 1 (Hsp70/Hsp90-organizing) stip1	V	P	−0.577	
U5KQ14	Heat shock protein 90-alpha 3	V	P	−0.659	[7,8,9]
B9ELQ1	Heat shock cognate 71 kDa protein	V	P	−1.01	
B5XGZ2	Glutathione S-transferase P	V	P	−2.25	[12]
U5KR11	Heat shock protein 90-alpha 4	V	P	−2.36	[7,8,9]
B5XDQ7	Heat shock factor-binding protein 1 HSBP1	V		V	
Q3ZLR1	Superoxide dismutase [Cu-Zn] SOD1	V		V	
Apoptosis					
B5X8Y9	Diablo homolog, mitochondrial	C	0.837	P	[7]
B5XB64	Thymocyte nuclear protein 1 THYN1	C	−1.92	P	
B9EL90	Programmed cell death protein 5	V		V	
B5DG91	Apoptosis-associated speck-like protein containing a CARD	V		V	
B5XDE1	Bcl10-interacting CARD protein		P	P	[7]
B5XEY3	Caspase-1		P	P	
B5XDX7	Programmed cell death 1 ligand 1 PDL1		P	P	
Cytoskeleton, organization and regulation					
B5DGE8	Tubulin alpha chain tuba8l2	3.84	2.56	−1.28	
C0HBL4	Tubulin beta-2A chain	3.20	1.51	−1.69	[7]
Q2ERI0	Beta-tubulin	3.12	1.95	−1.16	
A7KJD9	TUBB	3.09	1.98	−1.12	
B5X0U5	Tubulin beta chain	2.62	1.94	−0.676	
C0PU76	Tubulin alpha-1C chain	1.58	1.54	−0.0359	
B5DH01	Tubulin alpha chain TBA	1.55	1.80	0.251	
B5DH02	Tubulin, alpha 8 like 3-2	1.46	1.76	0.310	
C0H808	Tubulin beta-1 chain	1.42	0.766	−0.650	
B5XFE9	Tubulin alpha-1A chain	1.32	1.79	0.473	
B9EN30	Tubulin beta-2C chain	1.28	0.714	−0.568	
B5X3H7	Tubulin beta-3 chain	1.27	−0.500	−1.77	
B9EMD3	Tubulin-specific chaperone A	−1.01	0.290	1.30	
B5XFN3	Myosin light polypeptide 6B	1.40	−0.883	−2.28	
B5X9N0	Myosin light polypeptide 6	1.30	−0.837	−2.14	
C0PU50	Myosin-9	0.562	1.77	1.21	
B5XAM0	Thymosin beta-12	0.613	−1.83	−2.44	[7,9]
B5X2P7	Tropomyosin-1 alpha chain	−0.300	−0.118	0.182	
B5X2S2	Tropomyosin alpha-4 chain	−1.11	0.0879	1.20	[9]
B5X4C0	Tropomyosin alpha-3 chain	−1.52	0.023	1.55	[9]
B9ENC8	Transgelin TAGL	−1.02	−0.810	0.215	
B5X2M3	Septin-7	−1.16	−1.04	0.130	
B5XFZ3	Actin, adductor muscle	0.546	1.51	0.962	
B5X3R3	F-actin-capping protein subunit beta	0.890	−1.03	−1.92	
B5DG56	Capping protein (actin filament) muscle Z-line α 2	−0.277	−1.01	−0.732	
O42161	Actin, cytoplasmic 1	−0.516	−0.00485	0.511	[9]
B5DG40	Fast myotomal muscle actin 2	−0.917	0.154	1.07	
Q78BU2	Actin alpha 1-1	−0.967	0.104	1.07	
C0H9H2	Actin-related protein 2-A ARP2A	2.36	C	V	
B5DH12	Myosin light chain 1-1	2.02	C	V	
B5DGD5	Actin related protein 2/3 complex subunit 2 ARPC2	0.179	C	V	
B5XCW2	Actin-related protein 2/3 complex subunit 4 ARPC4	C	−2.76	P	
A8WCK1	Myosin 1	C	1.24	P	
C0HBJ2	Gamma-tubulin complex component 4	C	−0.519	P	
B5X3R2	Dynactin subunit 2	V	P	1.58	
C0HBE5	Actin-related protein 3 ARP3	V	P	−3.50	
C0H8E0	Actin-related protein 2/3 complex subunit 5 ARPC5	V		V	
B5RI24	Actinin alpha 3	V		V	
C0HBR6	Actin-related protein 2/3 complex subunit 1B ARC1B	V		V	
C0PUH9	Actin-related protein 2-B ARP2B	V		V	
B5XFD6	Myosin light polypeptide 4	V		V	
B5X1K8	Myosin regulatory light chain 2, smooth muscle isoform	V		V	
Q7ZZN0	Myosin regulatory light chain 2	V		V	
B5XG71	Actin-related protein 6 ARP6		P	P	
B5X2S3	Actin-related protein 8		P	P	
C0H9Q6	Rho GTPase-activating protein 15		P	P	
B5X3B9	Rho-related GTP-binding protein RhoE		P	P	
Lysosome, phagosome, vesicle fusion and trafficking					
B5XDV8	Cystatin-B	2.69	−3.43	−6.12	[12]
B5DFV6	Cathepsin D	−0.0872	C	V	[7,31]
C0H8J4	AP-1 complex subunit mu-2	C	0.923	P	[7]
B9ELB6	Charged multivesicular body protein 5 CHMP5	C	0.668	P	
C0HBD4	Interferon-induced guanylate-binding protein 1 GBP1	C	−0.188	P	[12,31]
C0H9C2	Phosphatidylinositol-5-phosphate 4-kinase type-2 beta	C	0.428	P	
B5X6D9	Ras-related protein ralB-B	C	−0.305	P	
B5X6L9	Ras-related protein Rab-39B	C	−1.12	P	
B5X127	Ras-related protein Rab-7a	V	P	0.251	
C0PUQ5	Cathepsin Z	V		V	[8]
B5XCD1	Vesicle-associated membrane protein-associated protein B	V		V	
C0PUP3	AP-2 complex subunit beta-1	V		V	[32]
B9EQ28	Clathrin light chain B	V		V	[32]
C0HAE8	Phosphatidylinositol-binding clathrin assembly protein PICA	V		V	
C0HAE8	Phosphatidylinositol-binding clathrin assembly protein	V		V	
B9ENL7	Ras-related protein Rab-5C	V		V	
B5XFU5	Adaptin ear-binding coat-associated protein 1		P	P	
B5X152	AP-3 complex subunit mu-1		P	P	
B5RI38	Cathepsin l-like		P	P	[12]
C0HA36	Endophilin-B2		P	P	
C0H980	Lysosome-associated membrane glycoprotein 1 LAMP1		P	P	
B5X7X4	Ragulator complex protein LAMTOR2		P	P	
B5X198	Ran GTPase-activating protein 1 RGP1		P	P	
C0PU44	Ras-related protein Rab-14		P	P	
B5X0U0	Ras-related protein Rab-18		P	P	
B5X481	Synaptotagmin-4 SYT4		P	P	
B5X179	Syntaxin-3		P	P	
C0H9V3	Syntaxin-5		P	P	
C0PUM9	Syntaxin-binding protein 2 STXB2		P	P	

## Supplementary Materials

The following are available online at https://www.mdpi.com/2076-2607/8/12/1845/s1, Figure S1: Schematic representation of sample pools obtained for global proteomic profiling of *P. salmonis* infections in SHK-1 cultures, Table S1: *P. salmonis* LF-89 and *S. salar* SHK-1 cells quantitative proteomics results, Table S2: Primers used for qPCR analyses, Table S3: Enrichment analyses of *S. salar* proteomic results, Table S4: qPCR analysis of *P. salmonis* selected virulence factors.
