# Supplementary material for: Global Proteomic Profiling of Piscirickettsia salmonis and Salmon Macrophage-Like Cells during Intracellular Infection
Source: Microorganisms. 2020 Nov 24;8(12):1845. doi: 10.3390/microorganisms8121845 (PMC7760863; doi:10.3390/microorganisms8121845)
Supplement: Supplementary file 1 [file microorganisms-08-01845-s001.zip › Supplementary material/Figure S1.docx]

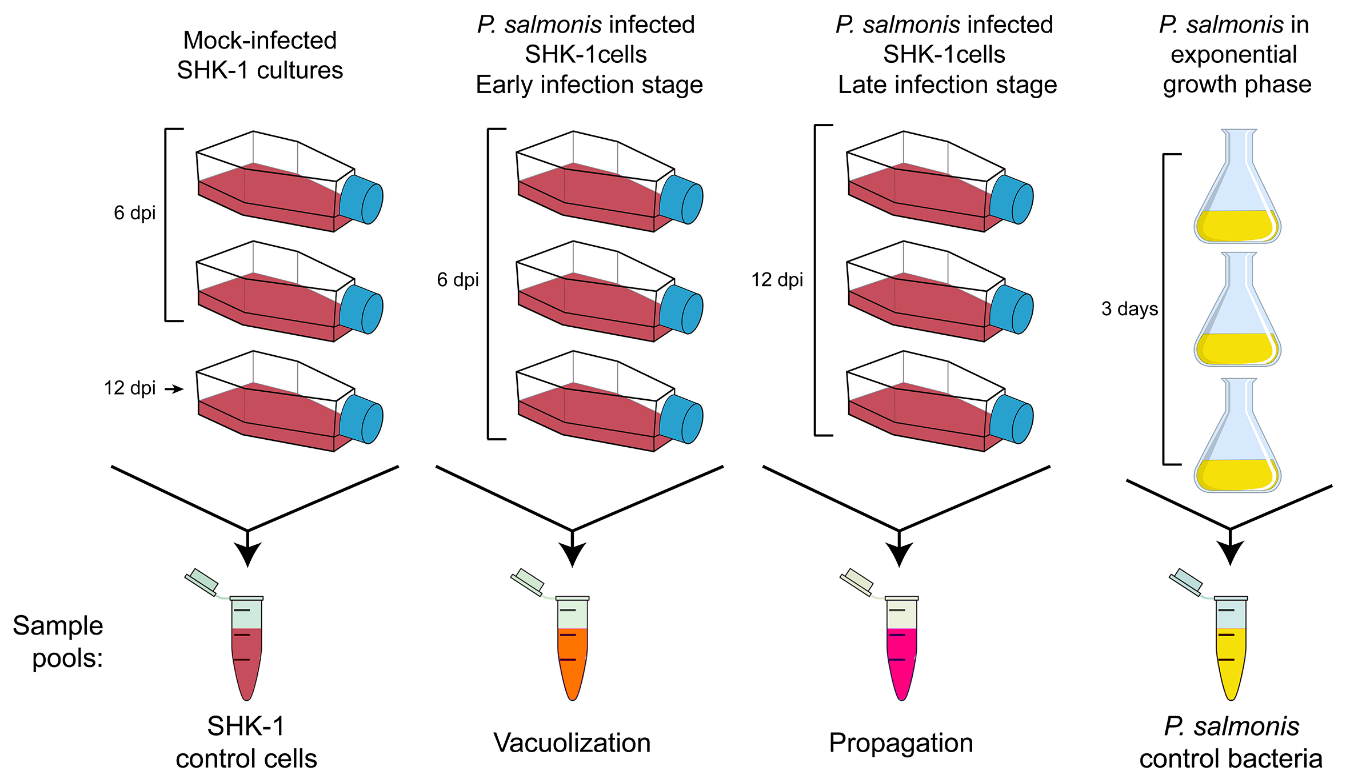


**Figure S1.** Schematic representation of sample pools obtained for global proteomic profiling of *P. salmonis* infections in SHK-1 cultures. Mock and *P. salmonis* infected SHK-1 cells were collected at early (6 days post-infection, vacuolization) and late (12 days post-infection, propagation) infection stages. *P. salmonis* grown in nutrient broth in exponential-growth phase was use as bacterial control.
